# Identification of Molecular Subtypes of B-Cell Acute Lymphoblastic Leukemia in Mexican Children by Whole-Transcriptome Analysis

**DOI:** 10.3390/ijms26147003

**Published:** 2025-07-21

**Authors:** Norberto Sánchez-Escobar, María de los Ángeles Romero-Tlalolini, Haydeé Rosas-Vargas, Elva Jiménez-Hernández, Juan Carlos Núñez Enríquez, Angélica Rangel-López, José Manuel Sánchez López, Daniela Rojo-Serrato, América Mariana Jasso Mata, Efraín Abimael Márquez Aguilar, Janet Flores-Lujano, Juan Carlos Bravata-Alcántara, Jorge Alfonso Martín-Trejo, Silvia Jiménez-Morales, José Arellano-Galindo, Aurora Medina Sanson, Jose Gabriel Peñaloza Gonzalez, Juan Manuel Mejía-Aranguré, Minerva Mata-Rocha

**Affiliations:** 1 SECIHTI-Facultad de Medicina y Cirugía-Universidad Autónoma “Benito Juárez” de Oaxaca, Mexico City 68020, Mexico; venancio.saes@gmail.com (N.S.-E.); romerotlalolini@gmail.com (M.d.l.Á.R.-T.); 2 Unidad de Investigación Médica en Genética Humana, Hospital de Pediatría “Dr. Silvestre Frenk Freund”, Centro Médico Nacional Siglo XXI, Instituto Mexicano del Seguro Social, Mexico City 06720, Mexico; hayrov@gmail.com (H.R.-V.); cienciaflosan@gmail.com (J.M.S.L.); danielarojo.serrato@gmail.com (D.R.-S.); americajassomata@gmail.com (A.M.J.M.); abimaelmarquez@gmail.com (E.A.M.A.); 3 Servicio de Oncohematología Pediátrica, Hospital Pediátrico Moctezuma, Secretaría de Salud de la Ciudad de México, Mexico City 15530, Mexico; elvajimenez@yahoo.com; 4 División de Investigación en Salud, Hospital de Pediatría “Dr. Silvestre Frenk Freund”, Centro Médico Nacional Siglo XXI, Instituto Mexicano del Seguro Social, Mexico City 06720, Mexico; jcarlos_nu@hotmail.com; 5 Laboratorio de Virología Clínica y Experimental, Unidad de Investigación en Enfermedades Infecciosas, Hospital Infantil de México Federico Gómez, Secretaría de Salud, Mexico City 06720, Mexico; angelica.rangell@imss.gob.mx (A.R.-L.); jose.arellano@salud.gob.mx (J.A.-G.); 6 Institute of Pharmacology and Structural Biology, 31077 Toulouse, France; 7 Escuela Superior de Medicina, Instituto Politécnico Nacional, Mexico City 11340, Mexico; 8 Unidad de Investigación Médica en Epidemiología Clínica, Hospital de Pediatría “Dr. Silvestre Frenk Freund”, Centro Médico Nacional Siglo XXI, Instituto Mexicano del Seguro Social, Mexico City 06720, Mexico; janetflores22@yahoo.com.mx; 9 Laboratorio de Genética y Diagnóstico Molecular, Hospital Juárez de México, Mexico City 07760, Mexico; juan.bravata@salud.gob.mx; 10 Servicio de Hematología, Hospital de Pediatría “Dr. Silvestre Frenk Freund”, Centro Médico Nacional Siglo XXI, Instituto Mexicano del Seguro Social, Mexico City 06720, Mexico; jorge.martin.trejo@gmail.com; 11 Laboratorio de Innovación y Medicina de Precisión, Núcleo “A”, Instituto Nacional de Medicina Genómica, Mexico City 14610, Mexico; sjimenez@inmegen.gob.mx; 12 Departamento de Oncología, Hospital Infantil de México Federico Gómez, Secretaría de Salud, Mexico City 06720, Mexico; auroramedina@gmail.com; 13 Servicio de Onco-Pediatría, Hospital Juárez de México, Secretaría de Salud, Mexico City 07760, Mexico; penaloza_6@yahoo.es; 14 Laboratorio de Genómica Funcional del Cáncer, Instituto Nacional de Medicina Genómica, Mexico City 14610, Mexico; 15 Facultad de Medicina, Universidad Nacional Autónoma de México, Mexico City 04360, Mexico; 16 SECIHTI-Unidad de Investigación Médica en Genética Humana, Hospital de Pediatría “Dr. Silvestre Frenk Freund”, Centro Médico Nacional Siglo XXI, Instituto Mexicano del Seguro Social, Mexico City 03940, Mexico

**Keywords:** molecular subtypes of B-ALL, RNA-seq, DUX4, transcriptome, NGS

## Abstract

B-lineage acute lymphoblastic leukemia (B-ALL) is classified into more than 20 molecular subtypes, and next-generation sequencing has facilitated the identification of these with high sensitivity. Bulk RNA-seq analysis of bone marrow was realized to identify molecular subtypes in Mexican pediatric patients with B-ALL. High hyperdiploidy (27.3%) was the most frequent molecular subtype, followed by *DUX4* (13.6%), *TCF3::PBX1* (9.1%), *ETV6::RUNX1* (9.1%), *Ph-like* (9.1%), *ETV6::RUNX1-like* (9.1%), *PAX5alt* (4.5%), Ph (4.5%), *KMT2A* (4.5%), and *ZNF384* (4.5%), with one patient presenting both the *PAX5alt* and low hypodiploidy subtypes (4.5%). The genes *TYK2, SEMA6A, FLT3, NRAS, SETD2, JAK2, NT5C2, RAG1*, and *SPATS2L* harbor deleterious missense variants across different B-ALL molecular subtypes. The *Ph-like* subtype exhibited mutations in *STAT2*, *ADGRF1*, *TCF3*, *BCR*, *JAK2*, and *NRAS* with overexpression of the *CRLF2* gene. The *DUX4* subtype showed mutually exclusive missense variants in the *PDGRFA* gene. Here, we have demonstrated the importance of using RNA-seq to facilitate the differential diagnosis of B-ALL with successful detection of gene fusions and mutations. This will aid both patient risk stratification and precision medicine.

## 1. Introduction

B-ALL is a malignant transformation and proliferation of B lymphoid progenitor cells in the bone marrow and is the most common childhood cancer [1]. In recent years, Mexico’s B-ALL incidence has increased to 53.1 during the period 2010–2017, compared with 49.5 during 2006–2007, in terms of cases per million in the population <15 years of age, as previously described by our group [2,3]. B-ALL is characterized by numerous pathogenic genomic lesions [4], and identifying these lesions has improved the molecular taxonomy of B-ALL in more than 20 different molecular subtypes, which are relevant to prognosis and targeted therapies [5,6,7,8]. For example, the *BCR::ABL1-like, KMT2A,* and *BCR::ABL1* subtypes have been associated with and defined by inferior survival [9,10]. Therefore, the identification of these molecular subtypes helps to predict a high risk of relapse and death.

The *ZNF384* subtype has been associated with a favorable prognosis when presenting the *ZNF384* rearrangement with *EP300*, *TAF15*, *EWSR1*, *ARID1B*, *TCF4*, *BMP2K*, or *CREBBP*, but has a less favorable prognosis with the *TCF3::ZNF384* rearrangement [11]. The *PAX5alt* subtype has an intermediate prognosis compared with those with a good prognosis such as the *ETV6::RUNX1* subtype, which presents high event-free survival rates [12]. The prognosis of the *ETV6::RUNX1-like* subtype is still controversial: it has been reported to have a good prognosis in studies with limited numbers of samples [13,14]. Additionally, for other molecular subtypes such as *MEF2D*-r, *PAX5P80R*, *NUTM1*-r, *ZEB2/CEBP*, *BCL2/MYC*, *IGH::ID4*, and *IGH::IL3*, it is necessary to carry out more work and expand the number of samples to define a better association with prognosis because the works published so far base their conclusions on a limited number of samples. In addition, the association of molecular subtypes of ALL with genetic ancestry reveals that Amerindians are associated with a low probability of subtypes *ETV6::RUNX1, BCR::ABL1, TCF3::PBX1,* and *KMT2A*, and show an increased propensity to other molecular subtypes such as *DUX4, PAX5alt,* and *ETV6::RUNX1-like* [15]. Therefore, the extensive study of B-ALL molecular subtypes will help to predict the risk of death or relapse, and the recognition of molecular subtypes will help refine risk stratification and optimize therapy [6,16,17,18,19].

However, determining a reliable molecular subtype is challenging due to the high genetic heterogeneity of each B-ALL subtype, and conventional tools such as fluorescence in situ hybridization (FISH), karyotyping, reverse transcriptase polymerase chain reaction (RT-PCR), and microarrays commonly used to detect genetic abnormalities are not sufficient to detect the high number of aberrations present [20]. Several articles have provided evidence that next-generation sequencing (NGS) is an important tool in identifying new and emerging molecular subtypes. RNA-seq technology has enabled the comprehensive genetic examination of patients with ALL, and has identified novel fusion genes and genetic variants such as *IKZF*1 deletion [14,21]. More importantly, the expression profile serves as a tool for clustering and determining molecular subtypes; therefore, its clinical use will become a powerful tool to predict prognostic outcomes and target therapy [22,23,24]. In this study, we obtained the whole transcriptomes of bone marrow samples from a cohort of Mexican pediatric patients diagnosed with B-ALL. We demonstrate the feasibility of using machine learning algorithms to identify a molecular subtype of B-ALL in a Mexican cohort.

## 2. Results

### 2.1. Clinical Characteristics of MIGICCL Cohort

**Figure 1:**
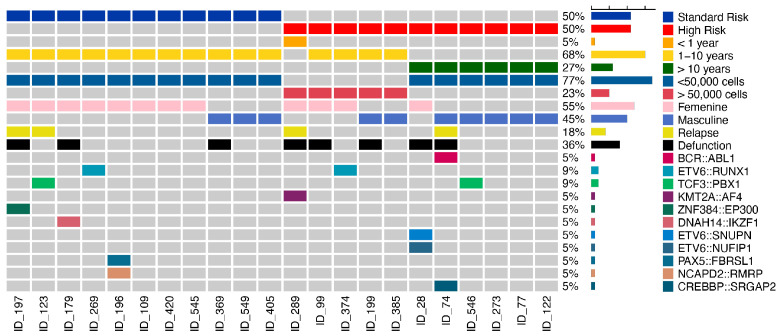
Oncoplot of distribution of clinical characteristics. The plot shows the frequency of the characteristics and outcome in each patient. Each column represents a patient with acute lymphoblastic leukemia (B-ALL) and each row represents a clinical characteristic corresponding to a patient; right bar represents the frequency of clinical characteristic.

Twenty-two patients diagnosed with B-ALL who had a mean age of 5 years (range 0.6–17 years) were included, of whom only 10 were male. According to NCI criteria, 50% (*n*  =  11) were classified as high-risk patients. Eight patients (ID_197, ID_289, ID_74, ID_369, ID_99, ID_179, ID_199 and ID_28), representing 36%, died; three of them (ID_197, ID_289, and ID_74) had previous relapses. In total, four B-ALL patients (ID_123, ID_197, ID_289, and ID_74) experienced a relapse (Figure 1). For the NLP controls, the mean age of patients was 4 years (range 2–7 years), and two were male.

### 2.2. Bioinformatic Determination of Genetic Fusions

The analysis of transcriptome sequencing data is difficult because repeated and short reads with similar sequences can result in ambiguous alignments, lowering the sensitivity and specificity. Therefore, we decided that we had to use more than one algorithm in order to detect all fusion events. Using the TopHat-Fusion and Manta algorithms, we identified 13 total gene fusions (11 unique) in 10 samples, which is more gene fusions than were revealed in the previous report [25]. The TopHat-Fusion algorithm in the first analysis allowed us to detect nine fusion events: *ETV6::SNUPN*, *ETV6::NUFIPN*, *ZNF384::EP300*, *IKZF1::DNAH14*, *CREBBP*::S*RGAP2*, *BCR::ABL1*, *KMT2A::AFF1*, *ETV6::RUNX1*, and *TCF3::PBX1.* In the second analysis, using Manta, we detected another two: *PAX5*::*FBRSL1* and *NCAPD2*::*RMRP*. Thus, between the two analyses, a total of 11 of these events were detected. The gene fusions identified are shown in Figure 1. Ten samples had at least one of these fusions and we found three patients, each with two gene fusions: ID_196 with *PAX5::FBRSL1* and *NCAPD2::RMRP*; ID_28 with *ETV6::SNUPN* and *ETV6::NUFIPN*; and ID_74 with *CREBBP*::*SRGAP2* and *BCR::ABL1 p190* (Figure 1).

### 2.3. Classification of B-ALL Molecular Subtypes by Machine Learning Classifiers

We constructed a heat map with the standard deviation of the expression profile, and the hierarchical cluster analysis showed similarities among expression patterns in B-ALL samples; clear differences were observed between B-ALL on the left samples and NLPs on the right in Supplementary Figure S1. Considering the gene expression profiles, we determined the molecular subtypes using three classifiers based on machine learning algorithms: ALLSorts, MD-ALL, and ALLCatchR. We used these three different algorithms based on expression levels to establish the molecular subtype of each patient in our cohort [26,27,28].

**Figure 2:**
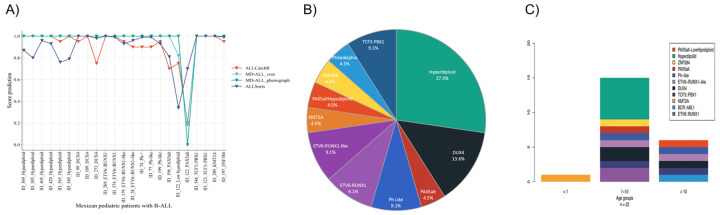
Prediction and frequency of molecular subtypes in Mexican children with ALL. (**A**) The line represents each algorithm used for prediction of our cohort. The X axis shows the score prediction from 0 to 1 and the Y axis the patients and their respective molecular subtype predicted. (**B**) The pie chart shows the frequency of molecular subtype in our cohort. (**C**) The age distribution of molecular subtype classified in three age ranges (<1, 1–10 and >10 years).

Positive predictive values or the full metrics of the machine learning models are available in Supplementary Table S1. All samples were assigned to one molecular subtype, as depicted in Figure 2A. The molecular subtypes named *ETV6::RUNX1, KMT2A, BCR::ABL1, TCF3::PBX1*, and *ZNF384::EP300* have a canonical fusion gene that defines them. The distributions of B-ALL molecular subtypes across cohorts and frequencies according to age are shown in Figure 2B. We found high hyperdiploidy in 27.3%, *DUX4* in 13.6%, *PAX5*alt in 4.5%, *TCF3::PBX1* in 9.1%, *ETV6::RUNX1* in 9.1%, *KMT2A* in 4.5%, *ETV6::RUNX1-*like in 9.1%, *BCR::ABL1* in 4.5%, Ph-like in 9.1%, *ZNF384* in 4.5%, and *PAX5alt*-low hypodiploidy in 4.5% of the patients (Figure 2B). The three algorithms agreed in assigning the molecular subtype for each sample—except for sample ID_122, which was classified by ALLSorts as the *PAX5alt* subtype with a 0.70 confidence score— differing from the MD-ALL and ALLCatchR algorithms’ low confidence scores of 0.18 and 0.2, respectively. The latter algorithms classified sample ID_122 as low hypodiploidy with scores of 0.34, 0.82 and 0.75 for ALLSorts MD-ALL and ALLCatchR, respectively.

### 2.4. Distributions of Different Molecular Subtypes of B-ALL

The frequencies of different B-ALL molecular subtypes in the MIGICCL cohort differed between age groups, with children >10 years of age harboring Ph, Ph-like, *PAX5alt*, *DUX4*, and *ETV6::RUNX1*-like subtypes, compared with children <10 years of age who comprised, additionally, the *ZNF384,* high hyperdiploidy, and *ETV6::RUNX1* subtypes (Figure 2C).

### 2.5. Gene Variants Associated with Molecular Subtypes

Biomarkers are important tools because they have prognostic significance and can be used for diagnosis and to aid early disease detection, risk stratification, and treatment guidance [20]. Therefore, we identified variants in genes previously reported in ALL by examining the sequencing reads in more detail, as RNA-seq also supports the detection of mutations and germline variations for hundreds to thousands of expressed transcripts and determines the allele-specific expression of these variants [29].

**Figure 3:**
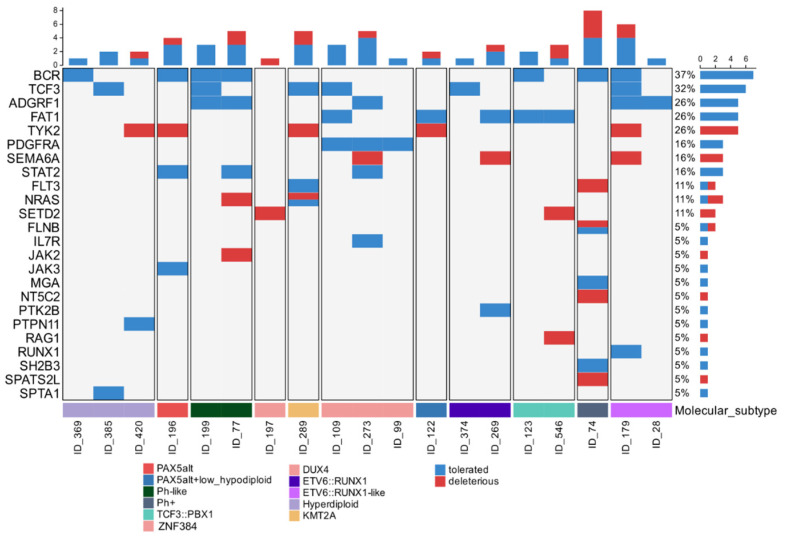
Oncoplot of the most frequently observed genes with mutations. Distribution of recurring missense variants across key genes implicated in acute lymphoblastic leukemia (ALL), classified as tolerated (blue) or deleterious (red) based on Sift Prediction. The oncoplot shows each column represents a patient (ID), and each row corresponds to a gene; the bar chart on the right shows the percentage of patients harboring variants in each gene. The plot is divided into molecular subtypes that are indicated by the color-coded labels below each column.

We analyzed the presence of missense variant (single-nucleotide variants [SNVs] and insertions/deletions [indels]) in genes that had already been reported to be altered in ALL. On average, we found 3 missense variants per patient (range 0–7). We identified *BCR* (37%), *TCF3* (32%), *ADGRF1* (26%), *FAT1* (26%), *TYK2* (26%), *PDGFRA* (38%), *SEMA6A* (16%), *STAT2* (16%), *FLT3* (11%), *NRAS* (11%) and *SETD2* (11%) recurrently with a missense variant that occurred across different B-ALL molecular subtypes and cannot be regarded as specific to any B-ALL molecular subtype; we only identified mutually exclusive missense variants in *PDGRFA* in the patients with the *DUX4* subtype (Figure 3). The genes *TYK2, SEMA6A, FLT3, NRAS, SETD2, JAK2, NT5C2, RAG1*, and *SPATS2L* harbor deleterious missense variants that are predicted to negatively affect the protein function or stability. Missense variants in non-receptor tyrosine kinases (NRTKs) were found in *TYK2* (26%), *PTK2B* (5%), *JAK2* (5%) and *JAK3* (5%) and were principally associated with the *Ph-like, PAX5alt*, and *ETV6::RUNX1* subtypes. Additionally, we identified key diagnostic mutations, such as *JAK2.* Arg683Gly was interpreted as pathogenic and reported in ClinVar ID 375951 in a patient with Ph-like subtype (ID_77), a known pathogenic somatic mutation (Figure 3).

### 2.6. Overexpression of CRFL2 Gene in Ph-like Subtype

Overexpression of the *CRLF2* (cytokine receptor-like factor 2) gene is an important characteristic of Ph-like subtype. In investigating the *CRFL2* expression levels, we figured out the median TPM (Transcripts Per Million) value for Ph-like and Non-Ph-like. As shown in Supplementary Figure S2A, the Ph-like mean TPM values range from 802 to 1995, and the Non-Ph-like values range from 62 to 95. For the verification of the *CRLF2* overexpression patterns, we used real-time quantitative RT-PCR validation. The *CRFL2* gene were assessed in Ph-like ID_199 and ID_77 and Non-Ph-like ID_273, ID_405_ID_74, ID_545, and ID_28 samples. *GAPDH* was used for the normalization of experiments. The data in Supplementary Figure S2B confirmed that Ph-like samples displayed the patterns align well with overexpression levels obtained from RNA-seq. The *CRFL2* overexpression levels of Ph-like were dramatically different from that of Non-Ph-like.

## 3. Discussion

Mexico has a high incidence and elevated mortality rates of childhood B-ALL, and the high risk of relapse or death (survival below 60%) [30] contrasts with the more-than 90% good outcomes in high-income countries [31]. This concerning difference could be explained by, among other factors, the inherent genetic factors associated with acute lymphoblastic leukemia risk development in the Hispanic population [32,33]. ALL is driven by different genetic alterations that define distinct molecular subtypes, such as the gene fusions formed by chromosomal rearrangements that are the main oncogenic drivers involved in the initiation and maintenance of leukemias and several defining molecular subtypes of B-ALL [34]. In the Mexican pediatric ALL population, the sum of the frequencies of common molecular subtypes (*BCR::ABL1*, *KMT2A::AFF1*, *ETV6::RUNX1*, and *TCF3::PBX1)* has been variously reported to be 24.2% [35], 17.7% [36], and 18.83% [37]. This is lower than in other countries: for example, it is 43.2% in South Korea [38] and 35% in the USA [20]. This reflects the compelling need to identify other B-ALL molecular subtypes in the Mexican population that are not diagnosed routinely. However, there is no consensus on the methods used to identify B-ALL molecular subtypes because each molecular subtype commonly has a large associated genetic abnormality. These are complex and will remain difficult to identify with common diagnostic techniques. Studies using NGS have helped define more than 20 molecular subtypes of B-ALL but the prognostic relevance of several of these subtypes is still not well established [7,8,21,22]. NGS is a powerful tool with broad applications. In the context of Leukemia, it can be utilized not only for defining molecular subtypes but also for accurately determining Minimal Residual Disease (MRD), which is crucial for risk stratification [39,40] and facilitates gene fusion analysis [41,42,43].

In our analysis of the Mexican cohort by RNA-seq, we found subtype-defining *BCR::ABL1*, *ETV6::RUNX1*, *KMT2A::AF1*, and *TCF3::PBX1*, *ZNF384::EP300* fusion transcripts that have been described as driver alterations [44,45,46,47,48], but also novel fusions *CREBBP::SRGAP2B*, *DNAH14::IKZF1*, *ETV6::SNUPN*, and *ETV6::NUFIP1,* which have not been reported previously in the existing databases or literature. In addition, the sequencing analysis revealed that the translocations that generate these fusions lead to a loss of *CREBBP* and *ETV6* coding sequences, and in *IKZF1* both the four zinc fingers and the dimerization domain are truncated [25]. Thus, the fusions affect these genes that encode transcription factors involved in B-cell differentiation and leukemogenesis [49].

In addition, we identified the B-ALL molecular subtype of the Mexican cohort using classifiers based on the relative analysis of gene expression profiles approach [50]. These classifiers are based on machine learning algorithms (MLAs) to enable a correct predictions of the molecular subtype of B-ALL [51]. MLAs are a subfield of artificial intelligence. Their application to these complex data will revolutionize diagnosis and treatment by identifying patterns and extracting insights that will significantly impact clinical decision-making [52]. MLAs have been applied in acute leukemia, including the identification of abnormal platelet counts as a significant risk in predicting pediatric ALL through classification and regression trees (CART), random forest (RM), gradient-boosted machine (GM), and C5.0 decision tree algorithms [53]. Machine learning (ML) classifiers have been developed to discern healthy B cells from lymphoblasts and classify stages of B-ALL [54], to predict cranial radiotherapy treatment in pediatric ALL patients [55], and to predict relapse of pediatric ALL based on clinical and laboratory data [56]. They have also been used to develop models for the accurate differential diagnosis of acute leukemia as *BCR::ABL p^190^* on the basis of specific gene expression data [57].

This is the first study in Mexico to report the frequency of ALL molecular subtypes in a Mexican cohort using RNA-seq and MLAs with high predictive confidence, reflecting the stability of the prediction from the classifiers. All samples were classified to a specific molecular subtype of B-ALL. Only patient ID_122 was classified as *PAX5alt* by ALLSorts and as low hypodiploidy by AllCatchR and MD-ALL algorithms. This could mean that this patient had undergone genetic modifications that led to the *PAX5alt* subtype and has a low hypodiploidy expression profile produced by numerical chromosomal aberrations. This could be similar to that reported for the low hypodiploidy subtype, which is related to the simultaneous transcriptional proximity to multiple subtypes, due to additional gene mutations [58].

A significant difference in the distributions of molecular subtypes according to age has been reported [59]. Comparing the distributions of subtypes among cohorts, we found that the prevalence of the *DUX4* subtype increased with age in patients with B-ALL, while *ETV6::RUNX1-like* was significantly more frequent in patients younger than 10 years. Both the *DUX4* and *ETV6::RUNX1 like* subtypes could be associated with a bad prognosis because these patients in the MIGICCL cohort died, differing from the *ETV6::RUNX1* subtype, related to good prognosis. It is important to highlight that this sample size limits the robustness of the conclusions regarding B-ALL in the Mexican population; therefore, subsequent analyses with a larger sample size may change the distribution of subtype proportions across different age groups.

The high hyperdiploidy is the most common molecular subtype in ALL and is associated with a good prognosis [60]. Even though the high hyperdiploidy subtype can be determined by conventional methods such as cytogenetics, many hospitals in Mexico routinely fail to test for it, due to a lack of resources. In this study, the high hyperdiploidy subtype accounted for approximately 27.3% of cases and was the most frequent molecular subtype in our cohort. In pediatric B-ALL the most frequent chromosomal lesion is *ETV6::RUNX1,* found in 25% of cases, but in Mexico it accounts for only 10.5% of cases [35]. This is possibly related to the Mexican population’s high degree of Native American genetic ancestry [15]. If the *ETV6::RUNX1* subtype is related to good prognosis and given that in Mexico this subtype is uncommon, it is very important to determine the other subtypes related to good prognosis to adjust chemotherapies, and this can help reduce the risk of side effects.

In relation to the *ETV6::RUNX1-like* subtype, *IKZF1* and *ETV6* are present as a rearrangement and we observed that these patients died; therefore, a thorough study of this subtype is required to define its real prognosis in the Mexican population. The second molecular subtype frequently observed was *DUX4*. One patient died; this poor outcome is contrary to the reported low risk of this subtype [61], but may also be due to the small sample size and therefore its prognosis needs to be investigated in future studies in Mexican patients. The missense variants in the *DUX4* subtype in our MIGICCL cohort are present in genes involved in transmembrane signaling receptors, transcription factors, membrane-bound signaling molecules and G-protein coupled receptors. Interestingly we found variants in the *PDGRFA* gene to be recurrent among the three patients with the *DUX4* subtype. However, analyses of variants in other populations did not reveal variants in *PDGRFA*; they detected variants for the *DUX4* subtype in *TBL1XR1, ERG, MYC, NCOR1, NRAS, ARID1B,* and *CTCF*, principally related to catalytic activity and transcriptional regulator activity [61,62,63,64,65]. This difference is probably due to ethnic composition related to genetic or possibly environmental factors in our population, as has been reported [66]. Because our study population was recruited from public hospitals of Mexico City, and therefore enriched for Hispanic patients, this population had worse prognoses than did Caucasians [67,68], but a study with more patients and a determination of ethnic compositions by genetic ancestry mapping is necessary to confirm this. *PDGFRA* is a proto-oncogene that plays an active role in activating cell signaling pathways essential for cell growth and differentiation [69]. *PDGFRA* is not normally expressed in bone marrow, except in stromal cells (but not in lymphoid precursors) [70]. Thus, it is important to study the expression of this gene in the *DUX4* subtype. Interestingly, PDGFRA proteins are reliable biomarkers of gastrointestinal stromal tumors (GISTs) that respond to overall survival improvement with imatinib therapy [71]. Likewise, kinase inhibitors that block *PDGFRA*—ripretinib, avapritinib, and crenolanib—are used, and different combinations of these drugs in GIST treatment have been proposed [72,73]. In the future, the effects of specific drugs on *PDGFRA* should be investigated. Alone or combined with conventional chemotherapies, they could be used to improve the prognosis of ALL patients with the *DUX4* subtype.

The Ph-like subtype is clinically highly relevant because it is characterized by diverse alterations leading to the activation of intracellular kinase signaling pathways (*ABL* and/or *JAK-STAT*) amenable to molecularly targeted therapies [74,75,76,77]. The worldwide incidence of the Ph-like subtype is 12% to 15% in children, increasing to 20% in adolescents, and significantly higher in adults [78]. Patients with this subtype have higher MRD levels than other patients with B-ALL, and it is more common in Hispanic patients [79]. In the Mexican population, a previous study identified this subtype using different methods and found a high frequency, 38.5%, of the Ph-like subtype [80], while in our results it was 9.1%. This difference could be due to the small sample size our cohort. Genetic alterations have been reported that are potential drug targets in the Ph-like subtype, so its detection is important. However, the determination requires various cytogenetic and molecular assays, which can be time-consuming and sometimes indeterminate due to technical limitations. We analyzed the Ph-like variants present in patients and found missense Arg683Gly in *JAK2,* a mutation located primarily in the pseudokinase domain of JAK2. It induces constitutive *JAK-STAT* activation that is abrogated with ruxolitinib (a *JAK* inhibitor) [81]. Overexpression of *CRFL2* mRNA in the Ph-like subtype is consistent with previous reports associating it with poor prognosis in B-ALL and the finding that the Ph-like subtype usually also harbors additional driving mutations [82]. *CRLF2* is located in the X and Y chromosome and in the pseudoautosomal region 1 (PAR1), and the CRLF2 protein joins to the IL-7 alpha receptor chain (IL7Rα) to form the high-affinity receptor for TSLP (Thymic Stromal Lymphopoietin). TSLP, upon binding to its receptor CRLF2, activates downstream pathways such as JAK-STAT and PI3K, positioning it as a crucial regulator of immune responses through cytokine signaling modulation. Also, TSLP drives Th2-polarized immunity by enhancing TCR-dependent T-cell proliferation and Th2 cytokine release, supporting B-cell expansion/differentiation, and amplifying Th2 signals from mast cells and natural killer T cells. Collectively, these actions position TSLP as a master regulator of Th2-mediated inflammation and regulate B-cell development [83]. Thus, RNA-seq can facilitate the identification of cases of Ph-like ALL and the reproducible detection of sequence variants through standardized algorithms with analytical validity. In contrast to conventional diagnostic methods requiring large numbers of cells, RNA-seq requires only one mcg of RNA. This is useful in cases where little material is available, particularly in infants, and shortens the time to diagnosis.

Although the low number of samples prevents us from extrapolating these findings to the Mexican pediatric population with ALL as a whole, this study begins to contribute to the understanding of B-ALL in the context of the Mexican genetic background, necessary to implement targeted therapies against the specific alterations present in Mexican patients.

## 4. Materials and Methods

### 4.1. MIGICCL Cohort

This study was conducted in accordance with the Declaration of Helsinki. Informed consent and samples were obtained from the parents of each child collected by the MIGICCL (Mexican Interinstitutional Group for the Identification of the Causes of Childhood Leukemia). Twenty-six bone marrow samples were collected from Mexican pediatric patients at diagnosis, including 22 with B-ALL and 4 nonleukemia patients (NLPs) who served as controls. Clinical details were obtained from medical records and a database was created. Follow-up information was also obtained for patients with ALL during treatment with modified therapeutic regimens, including St. Jude Total XIII (77.2%), Memorial Sloan-Kettering-New York-II (18.2%), and BFM-5 (4.6%). The diagnoses of the NLPs were hemophagocytic lymphohistiocytosis secondary to EBV (ID_73), mononucleosis IGM-positive for EBV (ID_159), EBV infection (ID_165), and bicytopenia (ID_83).

### 4.2. Bulk RNA-Seq

Using Lysis Solution (eBioscience, San Diego, CA, USA), red blood cell depletion from bone marrow was realized. Then, total RNA of white blood cells was extracted using a Direct-zol RNA kit (Zymo Research, Irvine, CA, USA). Libraries were synthesized using TruSeq Stranded Total RNA with Ribo-Zero Gold (Illumina, San Diego, CA, USA); briefly, rRNA was removed from total RNA then fragmented; and reverse transcriptase was used to synthesize cDNA and sequencing adapters were ligated. Libraries were evaluated using the 4200 TapeStation (Santa Clara, CA, USA) and sequenced for 2 X5 cycles (paired-end sequencing) on the Illumina sequencing platform (NextSeq500, Illumina, SanDiego, CA, USA), generating between 40 and 76 M reads per sample. We have published a first analysis of this raw data that consisted of the detection of novel fusions that were validated [25]. In this second report, we analyzed the raw data by using Gene Expression to investigate the molecular subtype of B-ALL.

### 4.3. Bioinformatic Analysis

Fastq files were trimmed for low quality reads using Trimmomatic, and aligned to the GRCh37 human genome using the STAR Aligner v 2.7.1. The quantification of the transcripts was realized with Salmon v1.10.2 and the counts were normalized EdgeR v3.17, for sequencing depth and gene length using the Transcripts Per Million (TPM) method. Fused genes were detected using TopHat-Fusion [26] and the Manta algorithm [27]. Variants were called with Strelka (Illumina) combined with manual curation. Strelka was utilized in its stringency settings to generate the list of putative synonymous, intron, splice, missense variants and the oncoplot was generated considering only missense variants. Manual filtration was conducted: include only variants with a minimum read depth of 50×, variants that were detected in NLP were excluded. And all identified somatic variant calls were examined by visual inspection of the BAM files of ALL and NLP samples by IGV. We used AllSorts [28], MD-ALL [29], and ALLCatchR [30] to determine the molecular subtype for each sample.

### 4.4. Validation of CRFL2 Expression by qRT-PCR

The fusion gene *CRLF2* expression was evaluated by qRT-PCR; for this purpose, we designed specific primers and probes, and we used GAPDH as an endogenous control transcript (Supplementary Table S2). QuantiNova Multiplex RT-PCR (QIAGEN) was employed according to the manufacturer’s instructions: briefly, a PCR reaction mixture was prepared containing 5 μL of 4× Master Mix, 10 µM of each primer, and 2.5 µM of CRFL2 and *GAPDH* TaqMan probes, 0.2 μL of 100× Multiplex Reverse Transcription Mix, and 100 ng of RNA from the corresponding sample, in a final volume of 20 μL. The cycler protocol was as follows: reverse transcription (50 °C for 10 min), activation, and initial DNA denaturation (95 °C for 2 min) for 1 cycle, then 95 °C for 5 s and 60 °C for 30 s for 40 cycles. The threshold cycles of the *CRFL2* and *GAPDH* genes were acquired in triplicate for each sample. The analysis was performed using ∆Ct, calculated as the difference between the Ct of *CRFL2* and the average Ct of *GAPDH*; ∆∆Ct was calculated from the difference among B-ALL patients and NLPs, and then the value of 2^−∆∆Ct^ was calculated.

## 5. Conclusions

In summary, in a cohort of Mexican children with ALL, RNA-seq precisely defined various molecular subtypes of B-ALL. We believe RNA-seq will be a first-line diagnostic method that will complement cytogenetic and molecular analyses in diagnosing ALL. However, translation into clinical practice remains challenging due to the need for infrastructure and personnel specialized in molecular biology.

## Data Availability

RNA-seq data generated in this study is available at NCBI BioProject database: https://www.ncbi.nlm.nih.gov/bioproject/?term=PRJNA809691. Access date: 23 February 2022.

## Supplementary Materials

The following supporting information can be downloaded at https://www.mdpi.com/article/10.3390/ijms26147003/s1.

## References

[1] Inaba H., Greaves M., Mullighan C.G. (2013). Acute lymphoblastic leukaemia. Lancet.

[2] Flores-Lujano J., Duarte-Rodriguez D.A., Jimenez-Hernandez E., Martin-Trejo J.A., Allende-Lopez A., Penaloza-Gonzalez J.G., Perez-Saldivar M.L., Medina-Sanson A., Torres-Nava J.R., Solis-Labastida K.A. (2022). Persistently high incidence rates of childhood acute leukemias from 2010 to 2017 in Mexico City: A population study from the MIGICCL. Front. Public Health.

[3] Perez-Saldivar M.L., Fajardo-Gutierrez A., Bernaldez-Rios R., Martinez-Avalos A., Medina-Sanson A., Espinosa-Hernandez L., Flores-Chapa Jde D., Amador-Sanchez R., Penaloza-Gonzalez J.G., Alvarez-Rodriguez F.J. (2011). Childhood acute leukemias are frequent in Mexico City: Descriptive epidemiology. BMC Cancer.

[4] Mullighan C.G. (2014). The genomic landscape of acute lymphoblastic leukemia in children and young adults. Hematol. Am. Soc. Hematol. Educ. Program.

[5] Davis K., Sheikh T., Aggarwal N. (2023). Emerging molecular subtypes and therapies in acute lymphoblastic leukemia. Semin. Diagn. Pathol..

[6] Ryan S.L., Peden J.F., Kingsbury Z., Schwab C.J., James T., Polonen P., Mijuskovic M., Becq J., Yim R., Cranston R.E. (2023). Whole genome sequencing provides comprehensive genetic testing in childhood B-cell acute lymphoblastic leukaemia. Leukemia.

[7] Arber D.A., Orazi A., Hasserjian R.P., Borowitz M.J., Calvo K.R., Kvasnicka H.M., Wang S.A., Bagg A., Barbui T., Branford S. (2022). International Consensus Classification of Myeloid Neoplasms and Acute Leukemias: Integrating morphologic, clinical, and genomic data. Blood.

[8] Alaggio R., Amador C., Anagnostopoulos I., Attygalle A.D., Araujo I.B.O., Berti E., Bhagat G., Borges A.M., Boyer D., Calaminici M. (2022). The 5th edition of the World Health Organization Classification of Haematolymphoid Tumours: Lymphoid Neoplasms. Leukemia.

[9] Heatley S.L., Sadras T., Kok C.H., Nievergall E., Quek K., Dang P., McClure B., Venn N., Moore S., Suttle J. (2017). High prevalence of relapse in children with Philadelphia-like acute lymphoblastic leukemia despite risk-adapted treatment. Haematologica.

[10] Gorecki M., Koziol I., Kopystecka A., Budzynska J., Zawitkowska J., Lejman M. (2023). Updates in KMT2A Gene Rearrangement in Pediatric Acute Lymphoblastic Leukemia. Biomedicines.

[11] Zhu L., Bai W., Cheng Q., Fang J. (2023). ZNF384-Related Fusion Genes in Acute Lymphoblastic Leukemia. Cancer Control.

[12] Qiu K.Y., Xu H.G., Luo X.Q., Mai H.R., Liao N., Yang L.H., Zheng M.C., Wan W.Q., Wu X.D., Liu R.Y. (2021). Prognostic Value and Outcome for ETV6/RUNX1-Positive Pediatric Acute Lymphoblastic Leukemia: A Report from the South China Children’s Leukemia Group. Front. Oncol..

[13] Zaliova M., Kotrova M., Bresolin S., Stuchly J., Stary J., Hrusak O., Te Kronnie G., Trka J., Zuna J., Vaskova M. (2017). ETV6/RUNX1-like acute lymphoblastic leukemia: A novel B-cell precursor leukemia subtype associated with the CD27/CD44 immunophenotype. Genes. Chromosomes Cancer.

[14] Lilljebjorn H., Henningsson R., Hyrenius-Wittsten A., Olsson L., Orsmark-Pietras C., von Palffy S., Askmyr M., Rissler M., Schrappe M., Cario G. (2016). Identification of ETV6-RUNX1-like and DUX4-rearranged subtypes in paediatric B-cell precursor acute lymphoblastic leukaemia. Nat. Commun..

[15] Lee S.H.R., Antillon-Klussmann F., Pei D., Yang W., Roberts K.G., Li Z., Devidas M., Yang W., Najera C., Lin H.P. (2022). Association of Genetic Ancestry with the Molecular Subtypes and Prognosis of Childhood Acute Lymphoblastic Leukemia. JAMA Oncol..

[16] Bastian L., Schroeder M.P., Eckert C., Schlee C., Tanchez J.O., Kampf S., Wagner D.L., Schulze V., Isaakidis K., Lazaro-Navarro J. (2019). PAX5 biallelic genomic alterations define a novel subgroup of B-cell precursor acute lymphoblastic leukemia. Leukemia.

[17] Li Z., Lee S.H.R., Chin W.H.N., Lu Y., Jiang N., Lim E.H., Coustan-Smith E., Chiew K.H., Oh B.L.Z., Koh G.S. (2021). Distinct clinical characteristics of DUX4- and PAX5-altered childhood B-lymphoblastic leukemia. Blood Adv..

[18] Almeida A.R.M., Neto J.L., Cachucho A., Euzebio M., Meng X., Kim R., Fernandes M.B., Raposo B., Oliveira M.L., Ribeiro D. (2021). Interleukin-7 receptor alpha mutational activation can initiate precursor B-cell acute lymphoblastic leukemia. Nat. Commun..

[19] Bastian L., Hartmann A.M., Beder T., Hanzelmann S., Kassens J., Bultmann M., Hoeppner M.P., Franzenburg S., Wittig M., Franke A. (2022). UBTF::ATXN7L3 gene fusion defines novel B cell precursor ALL subtype with CDX2 expression and need for intensified treatment. Leukemia.

[20] Zhang L., Habeebu S.S.M., Li W., Li W. (2022). Prognostic and Predictive Biomarkers in Precursor B-cell Acute Lymphoblastic Leukemia. Leukemia.

[21] Chouvarine P., Antic Z., Lentes J., Schroder C., Alten J., Bruggemann M., Carrillo-de Santa Pau E., Illig T., Laguna T., Schewe D. (2021). Transcriptional and Mutational Profiling of B-Other Acute Lymphoblastic Leukemia for Improved Diagnostics. Cancers.

[22] Walter W., Shahswar R., Stengel A., Meggendorfer M., Kern W., Haferlach T., Haferlach C. (2021). Clinical application of whole transcriptome sequencing for the classification of patients with acute lymphoblastic leukemia. BMC Cancer.

[23] Brown L.M., Lonsdale A., Zhu A., Davidson N.M., Schmidt B., Hawkins A., Wallach E., Martin M., Mechinaud F.M., Khaw S.L. (2020). The application of RNA sequencing for the diagnosis and genomic classification of pediatric acute lymphoblastic leukemia. Blood Adv..

[24] Yap K.L., Furtado L.V., Kiyotani K., Curran E., Stock W., McNeer J.L., Kadri S., Segal J.P., Nakamura Y., Le Beau M.M. (2017). Diagnostic evaluation of RNA sequencing for the detection of genetic abnormalities associated with Ph-like acute lymphoblastic leukemia (ALL). Leuk. Lymphoma.

[25] Mata-Rocha M., Rangel-Lopez A., Jimenez-Hernandez E., Morales-Castillo B.A., Gonzalez-Torres C., Gaytan-Cervantes J., Alvarez-Olmos E., Nunez-Enriquez J.C., Fajardo-Gutierrez A., Martin-Trejo J.A. (2019). Identification and Characterization of Novel Fusion Genes with Potential Clinical Applications in Mexican Children with Acute Lymphoblastic Leukemia. Int. J. Mol. Sci..

[26] Schmidt B., Brown L.M., Ryland G.L., Lonsdale A., Kosasih H.J., Ludlow L.E., Majewski I.J., Blombery P., Ekert P.G., Davidson N.M. (2022). ALLSorts: An RNA-Seq subtype classifier for B-cell acute lymphoblastic leukemia. Blood Adv..

[27] Hu Z., Jia Z., Liu J., Mao A., Han H., Gu Z. (2024). MD-ALL: An integrative platform for molecular diagnosis of B-acute lymphoblastic leukemia. Haematologica.

[28] Beder T., Hansen B.T., Hartmann A.M., Zimmermann J., Amelunxen E., Wolgast N., Walter W., Zaliova M., Antic Z., Chouvarine P. (2023). The Gene Expression Classifier ALLCatchR Identifies B-cell Precursor ALL Subtypes and Underlying Developmental Trajectories Across Age. Hemasphere.

[29] Byron S.A., Van Keuren-Jensen K.R., Engelthaler D.M., Carpten J.D., Craig D.W. (2016). Translating RNA sequencing into clinical diagnostics: Opportunities and challenges. Nat. Rev. Genet..

[30] Allemani C., Matsuda T., Di Carlo V., Harewood R., Matz M., Niksic M., Bonaventure A., Valkov M., Johnson C.J., Esteve J. (2018). Global surveillance of trends in cancer survival 2000-14 (CONCORD-3): Analysis of individual records for 37 513 025 patients diagnosed with one of 18 cancers from 322 population-based registries in 71 countries. Lancet.

[31] Ward Z.J., Yeh J.M., Bhakta N., Frazier A.L., Girardi F., Atun R. (2019). Global childhood cancer survival estimates and priority-setting: A simulation-based analysis. Lancet Oncol..

[32] de Smith A.J., Jimenez-Morales S., Mejia-Arangure J.M. (2023). The genetic risk of acute lymphoblastic leukemia and its implications for children of Latin American origin. Front. Oncol..

[33] Feng Q., de Smith A.J., Vergara-Lluri M., Muskens I.S., McKean-Cowdin R., Kogan S., Brynes R., Wiemels J.L. (2021). Trends in Acute Lymphoblastic Leukemia Incidence in the United States by Race/Ethnicity From 2000 to 2016. Am. J. Epidemiol..

[34] Schroder J., Kumar A., Wong S.Q. (2019). Overview of Fusion Detection Strategies Using Next-Generation Sequencing. Methods Mol. Biol..

[35] Mata-Rocha M., Rangel-Lopez A., Jimenez-Hernandez E., Nunez-Enriquez J.C., Morales-Castillo B.A., Sanchez-Escobar N., Sepulveda-Robles O.A., Bravata-Alcantara J.C., Najera-Cortes A.S., Perez-Saldivar M.L. (2022). Low Prevalence of ETV6::RUNX1 Fusion Gene in a Hispanic Population. Front. Pediatr..

[36] Bekker-Mendez V.C., Miranda-Peralta E., Nunez-Enriquez J.C., Olarte-Carrillo I., Guerra-Castillo F.X., Pompa-Mera E.N., Ocana-Mondragon A., Rangel-Lopez A., Bernaldez-Rios R., Medina-Sanson A. (2014). Prevalence of gene rearrangements in Mexican children with acute lymphoblastic leukemia: A population study-report from the Mexican Interinstitutional Group for the identification of the causes of childhood leukemia. BioMed Res. Int..

[37] Juarez-Avendano G., Luna-Silva N.C., Chargoy-Vivaldo E., Juarez-Martinez L.A., Martinez-Rangel M.N., Zarate-Ortiz N., Martinez-Valencia E., Lopez-Martinez B., Pelayo R., Balandran J.C. (2020). Poor Prognosis Biomolecular Factors Are Highly Frequent in Childhood Acute Leukemias From Oaxaca, Mexico. Technol. Cancer Res. Treat..

[38] Cho Y.U., Chi H.S., Park C.J., Jang S., Seo E.J. (2012). Rapid detection of prognostically significant fusion transcripts in acute leukemia using simplified multiplex reverse transcription polymerase chain reaction. J. Korean Med. Sci..

[39] Reyes-Barron C., Burack W.R., Rothberg P.G., Ding Y. (2017). Next-Generation Sequencing for Minimal Residual Disease Surveillance in Acute Lymphoblastic Leukemia: An Update. Crit. Rev. Oncog..

[40] Stefan A.I., Radu L.E., Jardan D., Colita A. (2025). The emerging role of next-generation sequencing in minimal residual disease assessment in acute lymphoblastic leukemia: A systematic review of current literature. Front. Med..

[41] Heyer E.E., Blackburn J. (2020). Sequencing Strategies for Fusion Gene Detection. Bioessays.

[42] Liu S.V., Nagasaka M., Atz J., Solca F., Mullauer L. (2025). Oncogenic gene fusions in cancer: From biology to therapy. Signal Transduct. Target. Ther..

[43] Hasty P., Montagna C. (2014). Chromosomal Rearrangements in Cancer: Detection and potential causal mechanisms. Mol. Cell. Oncol..

[44] Kim J.C., Chan-Seng-Yue M., Ge S., Zeng A.G.X., Ng K., Gan O.I., Garcia-Prat L., Flores-Figueroa E., Woo T., Zhang A.X.W. (2023). Transcriptomic classes of BCR-ABL1 lymphoblastic leukemia. Nat. Genet..

[45] Jakobczyk H., Jiang Y., Debaize L., Soubise B., Avner S., Serandour A.A., Rouger-Gaudichon J., Rio A.G., Carroll J.S., Raslova H. (2022). ETV6-RUNX1 and RUNX1 directly regulate RAG1 expression: One more step in the understanding of childhood B-cell acute lymphoblastic leukemia leukemogenesis. Leukemia.

[46] Bursen A., Schwabe K., Ruster B., Henschler R., Ruthardt M., Dingermann T., Marschalek R. (2010). The AF4.MLL fusion protein is capable of inducing ALL in mice without requirement of MLL.AF4. Blood.

[47] Lin C.H., Wong S.H., Kurzer J.H., Schneidawind C., Wei M.C., Duque-Afonso J., Jeong J., Feng X., Cleary M.L. (2018). SETDB2 Links E2A-PBX1 to Cell-Cycle Dysregulation in Acute Leukemia through CDKN2C Repression. Cell Rep..

[48] Dickerson K.M., Qu C., Gao Q., Iacobucci I., Gu Z., Yoshihara H., Backhaus E.A., Chang Y., Janke L.J., Xu B. (2022). ZNF384 Fusion Oncoproteins Drive Lineage Aberrancy in Acute Leukemia. Blood Cancer Discov..

[49] Fischer U., Yang J.J., Ikawa T., Hein D., Vicente-Duenas C., Borkhardt A., Sanchez-Garcia I. (2020). Cell Fate Decisions: The Role of Transcription Factors in Early B-cell Development and Leukemia. Blood Cancer Discov..

[50] Cieslik M., Chinnaiyan A.M. (2018). Cancer transcriptome profiling at the juncture of clinical translation. Nat. Rev. Genet..

[51] Kumar Y., Koul A., Singla R., Ijaz M.F. (2023). Artificial intelligence in disease diagnosis: A systematic literature review, synthesizing framework and future research agenda. J. Ambient. Intell. Humaniz. Comput..

[52] Choon Y.W., Choon Y.F., Nasarudin N.A., Al Jasmi F., Remli M.A., Alkayali M.H., Mohamad M.S. (2023). Artificial intelligence and database for NGS-based diagnosis in rare disease. Front. Genet..

[53] Mahmood N., Shahid S., Bakhshi T., Riaz S., Ghufran H., Yaqoob M. (2020). Identification of significant risks in pediatric acute lymphoblastic leukemia (ALL) through machine learning (ML) approach. Med. Biol. Eng. Comput..

[54] Ayyappan V., Chang A., Zhang C., Paidi S.K., Bordett R., Liang T., Barman I., Pandey R. (2020). Identification and Staging of B-Cell Acute Lymphoblastic Leukemia Using Quantitative Phase Imaging and Machine Learning. ACS Sens..

[55] Kashef A., Khatibi T., Mehrvar A. (2020). Prediction of Cranial Radiotherapy Treatment in Pediatric Acute Lymphoblastic Leukemia Patients Using Machine Learning: A Case Study at MAHAK Hospital. Asian Pac. J. Cancer Prev..

[56] Pan L., Liu G., Lin F., Zhong S., Xia H., Sun X., Liang H. (2017). Machine learning applications for prediction of relapse in childhood acute lymphoblastic leukemia. Sci. Rep..

[57] Lee J., Cho S., Hong S.E., Kang D., Choi H., Lee J.M., Yoon J.H., Cho B.S., Lee S., Kim H.J. (2021). Integrative Analysis of Gene Expression Data by RNA Sequencing for Differential Diagnosis of Acute Leukemia: Potential Application of Machine Learning. Front. Oncol..

[58] Makinen V.P., Rehn J., Breen J., Yeung D., White D.L. (2022). Multi-Cohort Transcriptomic Subtyping of B-Cell Acute Lymphoblastic Leukemia. Int. J. Mol. Sci..

[59] Harrison C.J. (2013). Targeting signaling pathways in acute lymphoblastic leukemia: New insights. Hematol. Am. Soc. Hematol. Educ. Program.

[60] Vojcek A., Pajor L. (2018). High hyperdiploid acute lymphoblastic leukemia is a highly curable subtype of childhood leukemia. Magy. Onkol..

[61] Li J.F., Dai Y.T., Lilljebjorn H., Shen S.H., Cui B.W., Bai L., Liu Y.F., Qian M.X., Kubota Y., Kiyoi H. (2018). Transcriptional landscape of B cell precursor acute lymphoblastic leukemia based on an international study of 1,223 cases. Proc. Natl. Acad. Sci. USA.

[62] Zhang J., McCastlain K., Yoshihara H., Xu B., Chang Y., Churchman M.L., Wu G., Li Y., Wei L., Iacobucci I. (2016). Deregulation of DUX4 and ERG in acute lymphoblastic leukemia. Nat. Genet..

[63] Brady S.W., Roberts K.G., Gu Z., Shi L., Pounds S., Pei D., Cheng C., Dai Y., Devidas M., Qu C. (2022). The genomic landscape of pediatric acute lymphoblastic leukemia. Nat. Genet..

[64] Schwab C., Cranston R.E., Ryan S.L., Butler E., Winterman E., Hawking Z., Bashton M., Enshaei A., Russell L.J., Kingsbury Z. (2023). Integrative genomic analysis of childhood acute lymphoblastic leukaemia lacking a genetic biomarker in the UKALL2003 clinical trial. Leukemia.

[65] Krali O., Marincevic-Zuniga Y., Arvidsson G., Enblad A.P., Lundmark A., Sayyab S., Zachariadis V., Heinaniemi M., Suhonen J., Oksa L. (2023). Multimodal classification of molecular subtypes in pediatric acute lymphoblastic leukemia. NPJ Precis. Oncol..

[66] Duarte-Rodriguez D.A., Flores-Lujano J., McNally R.J.Q., Perez-Saldivar M.L., Jimenez-Hernandez E., Martin-Trejo J.A., Espinoza-Hernandez L.E., Medina-Sanson A., Paredes-Aguilera R., Merino-Pasaye L.E. (2024). Evidence of spatial clustering of childhood acute lymphoblastic leukemia cases in Greater Mexico City: Report from the Mexican Inter-Institutional Group for the identification of the causes of childhood leukemia. Front. Oncol..

[67] Acharya S., Hsieh S., Shinohara E.T., DeWees T., Frangoul H., Perkins S.M. (2016). Effects of Race/Ethnicity and Socioeconomic Status on Outcome in Childhood Acute Lymphoblastic Leukemia. J. Pediatr. Hematol. Oncol..

[68] Gomez-Almaguer D., Ruiz-Arguelles G.J., Ponce-de-Leon S. (1998). Nutritional status and socio-economic conditions as prognostic factors in the outcome of therapy in childhood acute lymphoblastic leukemia. Int. J. Cancer Suppl..

[69] Wu E., Palmer N., Tian Z., Moseman A.P., Galdzicki M., Wang X., Berger B., Zhang H., Kohane I.S. (2008). Comprehensive dissection of PDGF-PDGFR signaling pathways in PDGFR genetically defined cells. PLoS ONE.

[70] Li H., Ghazanfari R., Zacharaki D., Ditzel N., Isern J., Ekblom M., Mendez-Ferrer S., Kassem M., Scheding S. (2014). Low/negative expression of PDGFR-alpha identifies the candidate primary mesenchymal stromal cells in adult human bone marrow. Stem Cell Rep..

[71] Artinyan A., Kim J., Soriano P., Chow W., Bhatia S., Ellenhorn J.D. (2008). Metastatic gastrointestinal stromal tumors in the era of imatinib: Improved survival and elimination of socioeconomic survival disparities. Cancer Epidemiol. Biomark. Prev..

[72] Sun Y., Yue L., Xu P., Hu W. (2022). An overview of agents and treatments for PDGFRA-mutated gastrointestinal stromal tumors. Front. Oncol..

[73] Bauer S., George S., von Mehren M., Heinrich M.C. (2021). Early and Next-Generation KIT/PDGFRA Kinase Inhibitors and the Future of Treatment for Advanced Gastrointestinal Stromal Tumor. Front. Oncol..

[74] Pui C.H., Roberts K.G., Yang J.J., Mullighan C.G. (2017). Philadelphia Chromosome-like Acute Lymphoblastic Leukemia. Clin. Lymphoma Myeloma Leuk..

[75] Roberts K.G., Morin R.D., Zhang J., Hirst M., Zhao Y., Su X., Chen S.C., Payne-Turner D., Churchman M.L., Harvey R.C. (2012). Genetic alterations activating kinase and cytokine receptor signaling in high-risk acute lymphoblastic leukemia. Cancer Cell.

[76] Maude S.L., Tasian S.K., Vincent T., Hall J.W., Sheen C., Roberts K.G., Seif A.E., Barrett D.M., Chen I.M., Collins J.R. (2012). Targeting JAK1/2 and mTOR in murine xenograft models of Ph-like acute lymphoblastic leukemia. Blood.

[77] Roberts K.G., Yang Y.L., Payne-Turner D., Lin W., Files J.K., Dickerson K., Gu Z., Taunton J., Janke L.J., Chen T. (2017). Oncogenic role and therapeutic targeting of ABL-class and JAK-STAT activating kinase alterations in Ph-like ALL. Blood Adv..

[78] Jain N., Roberts K.G., Jabbour E., Patel K., Eterovic A.K., Chen K., Zweidler-McKay P., Lu X., Fawcett G., Wang S.A. (2017). Ph-like acute lymphoblastic leukemia: A high-risk subtype in adults. Blood.

[79] Roberts K.G., Pei D., Campana D., Payne-Turner D., Li Y., Cheng C., Sandlund J.T., Jeha S., Easton J., Becksfort J. (2014). Outcomes of children with BCR-ABL1-like acute lymphoblastic leukemia treated with risk-directed therapy based on the levels of minimal residual disease. J. Clin. Oncol. Off. J. Am. Soc. Clin. Oncol..

[80] Martinez-Anaya D., Moreno-Lorenzana D., Reyes-Leon A., Juarez-Figueroa U., Dean M., Aguilar-Hernandez M.M., Rivera-Sanchez N., Garcia-Islas J., Vieyra-Fuentes V., Zapata-Tarres M. (2022). Characterization of Philadelphia-like Pre-B Acute Lymphoblastic Leukemia: Experiences in Mexican Pediatric Patients. Int. J. Mol. Sci..

[81] Bercovich D., Ganmore I., Scott L.M., Wainreb G., Birger Y., Elimelech A., Shochat C., Cazzaniga G., Biondi A., Basso G. (2008). Mutations of JAK2 in acute lymphoblastic leukaemias associated with Down’s syndrome. Lancet.

[82] Chiaretti S., Brugnoletti F., Messina M., Paoloni F., Fedullo A.L., Piciocchi A., Elia L., Vitale A., Mauro E., Ferrara F. (2016). CRLF2 overexpression identifies an unfavourable subgroup of adult B-cell precursor acute lymphoblastic leukemia lacking recurrent genetic abnormalities. Leuk. Res..

[83] He R., Geha R.S. (2010). Thymic stromal lymphopoietin. Ann. N. Y. Acad. Sci..

