# Supplementary material for: Identification of Molecular Subtypes of B-Cell Acute Lymphoblastic Leukemia in Mexican Children by Whole-Transcriptome Analysis
Source: Int J Mol Sci. 2025 Jul 21;26(14):7003. doi: 10.3390/ijms26147003 (PMC12295331; doi:10.3390/ijms26147003)
Supplement: Supplementary file 1 [file ijms-26-07003-s001.zip › Supplementary_Figure_S1.pdf]

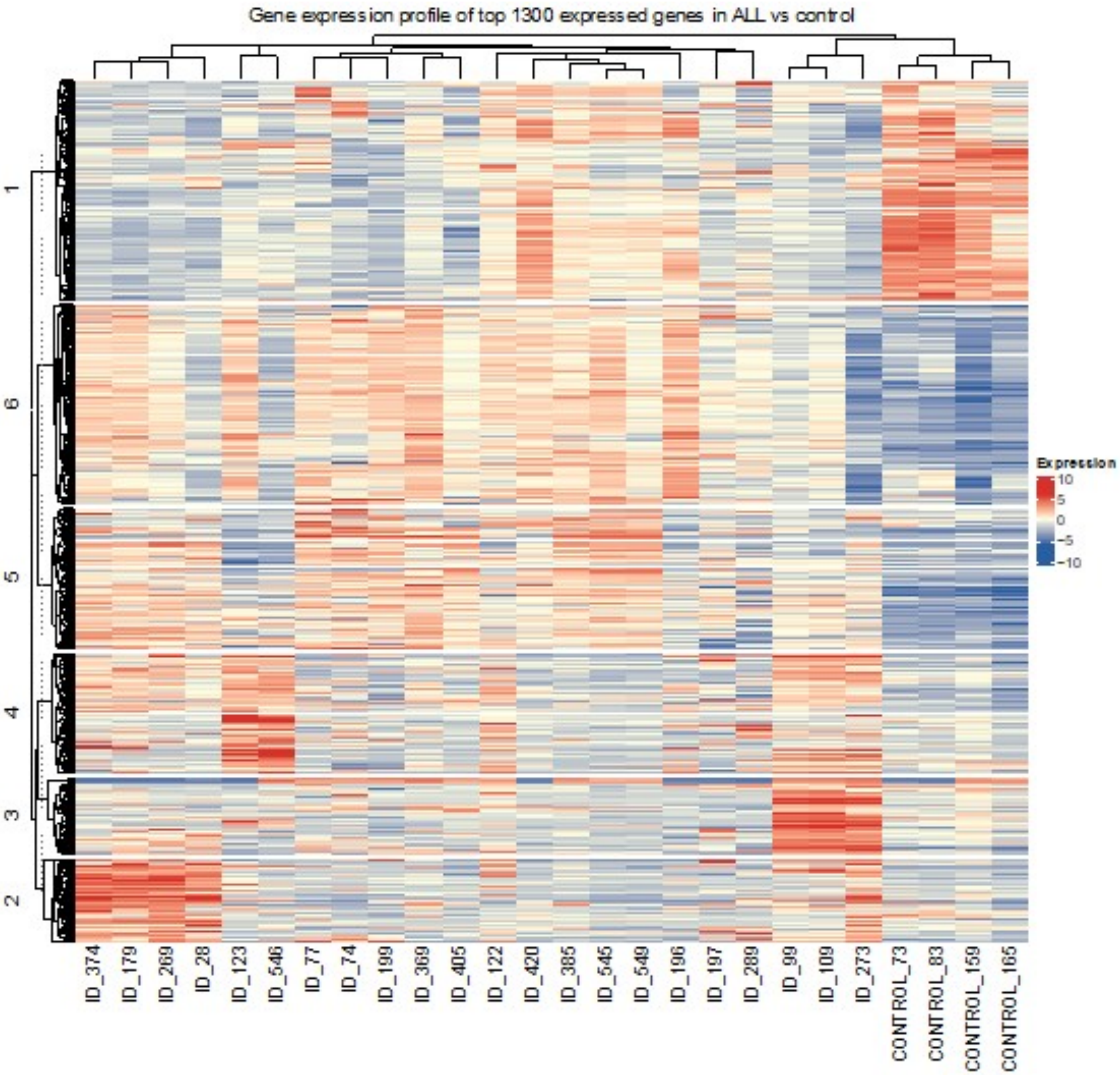

Supplementary figure 1. Heat map shows the expression profiles of one thousand three hundred genes that are differentially expressed in a sample of bone marrow with ALL. Each column is the profile of an individual patient, each row represents the abundance of one transcript from very low (dark blue) to low (light blue) to very high (dark red) expression.
CONTROL: nonleukemia patients (NLPs)
