# Supplementary material for: Identification of Molecular Subtypes of B-Cell Acute Lymphoblastic Leukemia in Mexican Children by Whole-Transcriptome Analysis
Source: Int J Mol Sci. 2025 Jul 21;26(14):7003. doi: 10.3390/ijms26147003 (PMC12295331; doi:10.3390/ijms26147003)
Supplement: Supplementary file 1 [file ijms-26-07003-s001.zip › Supplementary_Figure_S2.pdf]

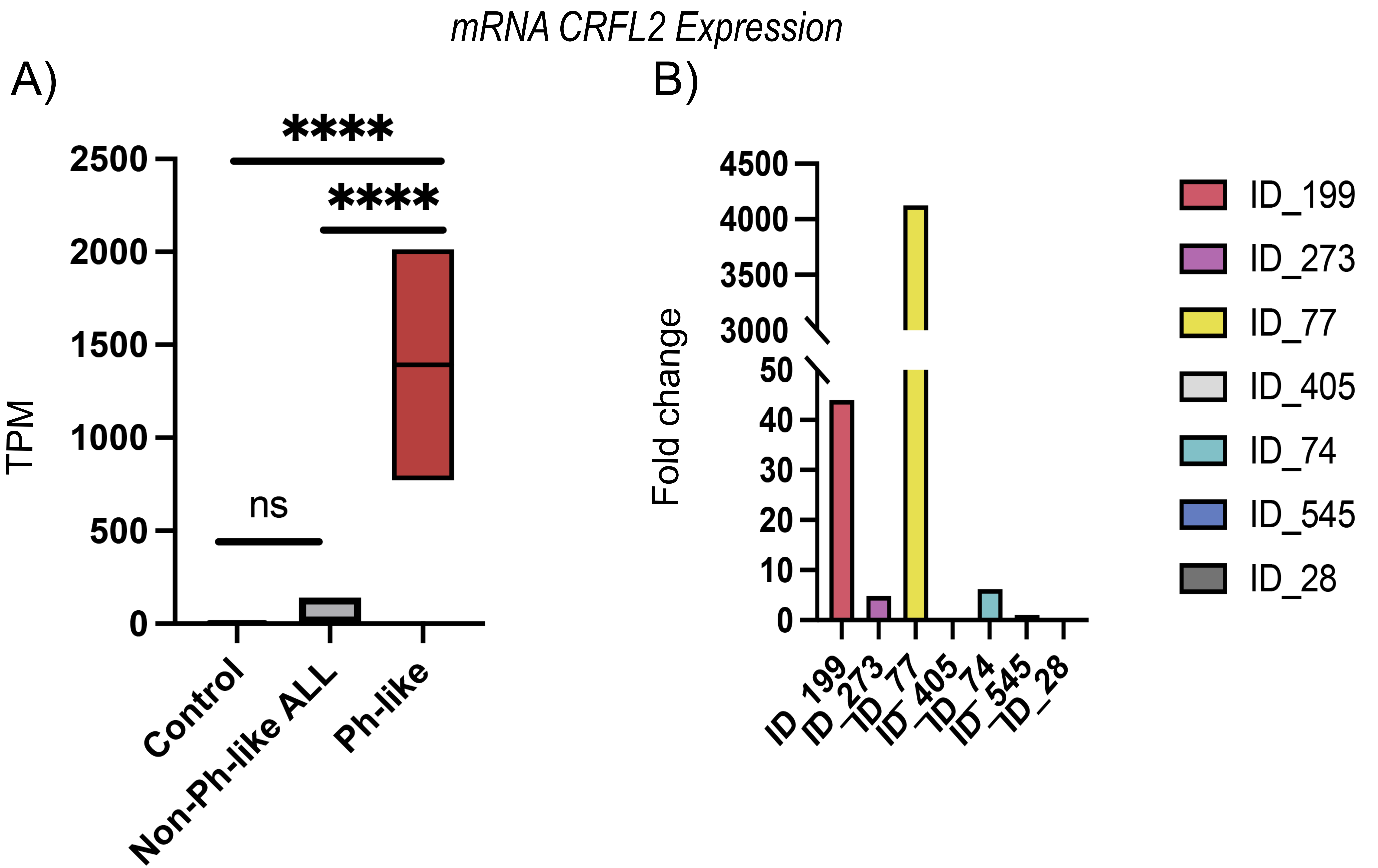

Supplementary Figure 2. High mRNA *CRFL2* Expression in Ph-like subtype. A) Comparison of normalized gene expressed levels (TPM) from RNAseq values for Ph-like and non-Ph-like subtype for *CRFL2* gene; ***p-value < 0.001; ns: not significant. **B)** Fold change of gene expression of *CRFL2* gene. qRT-PCR for mRNA expression of *CRLF2* in patients with B-ALL. Control: nonleukemia patients (NLPs)
